# 74123 A Learning Health Systems approach using health record data to construct patient frailty scores and predict safety events

**DOI:** 10.1017/cts.2021.528

**Published:** 2021-03-30

**Authors:** Alex Bokov, Sara Espinoza, Chandana Tripathy, Kathleen Stevens

**Affiliations:** UT Health San Antonio

## Abstract

ABSTRACT IMPACT: Laying the groundwork for better predictive algorithms to inform clinical decisions and planning. OBJECTIVES/GOALS: Frailty scores predict poor patient outcomes. Validated against highly relevant outcomes, such scores can be used to inform clinical and resource utilization decisions. We generated and validated an electronic Frailty Index (EFI) from real-world EHR data using the Rockwood deficit-accumulation framework to predict patient safety events. METHODS/STUDY POPULATION: To assure that the research approach reflected perspectives of multiple stakeholders, our multidisciplinary group included an implementation scientist, a geriatrician, an internist, and an informatician. From our large academic health center, we accessed EHR data for 14,844 patients randomly sampled from the data warehouse underlying our ACT/SHRINE node. The per-visit EFI scores were calculated using EHR codes in a rolling 2-year time window. EFI was used as the predictor variable in the analytic design. The primary outcomes were preventable patient-safety events derived from ICD-10 codes including hospital-acquired infections, non-operative hospital-acquired trauma, and cardiac complications. Cox proportional hazard models were used to estimate risk for each outcome. RESULTS/ANTICIPATED RESULTS: We found statistically significant associations of EFI with clinically meaningful outcomes from EHR data. For most outcomes, we found significant correlation with EFI and c-statistics indicating good calibration of the models. The EFI was a strong predictor of clinically relevant outcomes without relying on any data other than diagnoses, vital signs, and laboratory results from the EHR. In contrast to previous studies, we treated EFI as a time-varying predictor with multiple follow-ups per patient, which is more realistic than relying on one static time-point. We used a representative sample of the adult patient population rather than limiting it to older individuals and found EFI to be a useful metric even at relatively young ages. DISCUSSION/SIGNIFICANCE OF FINDINGS: The EFI predicted safety events in adult patients using only routine, structured EHR data and can offer a low-effort, scalable method of risk assessment, valuable to clinical decisions. The capability to harness EHR data and rapidly generate clinical knowledge can be transformative for complex care and contributes to Learning Health Systems.

